# Effects of Elastic Band Exercise on Functional Fitness and Blood Pressure Response in the Healthy Elderly

**DOI:** 10.3390/ijerph17197144

**Published:** 2020-09-29

**Authors:** Hyun-Min Choi, Chansol Hurr, Sukwon Kim

**Affiliations:** 1 Department of Sports Science, Gwangju University, 277, Gwangju-si 61743, Korea; hmchoi@gwangju.ac.kr; 2 Department of Physical Education, Jeonbuk National University, 567, Jeonju-si, Jeollabuk-do 54896, Korea

**Keywords:** elastic band exercise, functional fitness, blood pressure response, circuit training, elderly

## Abstract

*Purpose*: This study investigated the effects of 12 weeks of moderate intensity elastic band exercise (EBE) on functional fitness and blood pressure parameters in the elderly. *Methods*: 27 healthy older adults were randomly assigned to an exercise group (*n* = 15, age: 75.1 ± 1.4 years) and a control group (*n* = 12, age: 72.3 ± 1.4 years). Participants performed EBE for 60 min, three times a week, over the course of three months. The EBE consisted of incremental resistance and aerobic exercises designed to improve whole body fitness. Functional fitness and resting cardiovascular parameters were assessed before and after the exercise training program. *Results*: Grip strength, sit and reach, and one-leg stance improved significantly in the exercise group, while no significant improvements were found in chair stand and timed up and go (*p* < 0.05). Cardiovascular parameters including systolic blood pressure, diastolic blood pressure, mean arterial pressure, and pulse pressure significantly decreased in the exercise group relative to the control group (*p* < 0.05). *Conclusions*: Findings of the present study suggest that safe, inexpensive, and easily accessible EBE program with circuit training components play a significant role in improving upper and lower body fitness, as well as cardiovascular fitness, in the elderly.

## 1. Introduction

Individuals over 65 years old make up 14% of South Korea’s total population, and this is expected to increase to 20.8% by 2026, 24.3% by 2030, and 32.3% by 2040 [1]. Accordingly, the health problems associated with poor physical condition and disease are among the most serious issues in South Korea.

As one grows older, age-related changes in the body result in a simultaneous decline in physical activities in daily life [2,3,4]. This increases the risk of chronic diseases such as heart disease, arthritis, backache, neuralgia, high blood pressure, diabetes, and gastrointestinal diseases [2,3,4,5]. Insufficient physical activity and lack of exercise are the main causes of health problems in the elderly. Therefore, it is critical to develop a preventive plan that incorporates physical movement into one’s daily life.

The American College of Sports Medicine (ACSM) recommends aerobic and resistance exercise for older individuals [5]. Particularly, aerobic exercise decreases body fat, resting heart rate, and blood pressure [6], while resistance exercise is more effective at improving basal metabolic rate (BMR), bone density, and preventing sarcopenia [7]. Resistance exercises also improve muscular strength, balance, and walking speed in the elderly [8]. The resistance exercise, however, relies on large weight machines, such that exercises are only available at specific places. In this line, elastic bands could be effective, convenient, and cost-efficient tools for resistance exercise training for the elderly [9].

For elderly adults with diminished physical capabilities, resistance exercises using an elastic band increase muscular strength and fat free mass, and improve the physical functions of flexibility, ambulatory ability, static and dynamic balances, and backwards walking [10,11,12]. Previous studies have also shown that resistance exercises with moderate intensity improve endothelial function in blood vessels, and reduce arterial contraction, which decreases blood pressure and arterial stiffness [13]. Furthermore, trainers can tailor an exercise programs that accounts for the tension of the elastic band, length of the exercise session, the area of the body targeted (muscles, tendons, and ligaments) [14].

Although resistance and aerobic exercises are a useful intervention that improves muscular strength, body composition, and cardiovascular fitness in older adults, adherence to exercise programs among this population is quite low, as only ~22% of older adults participate in regular exercise activity at least twice a week [15]. Adherence to an exercise training program is dependent on program accessibility, exercise intensity, and frequency [16,17]. Circuit exercise training is physically less challenging and requires only 30~45 min for each session [18]. As a result, circuit exercise training, when compared with conventional resistance or aerobic exercise training [19], can promote higher adherence while improving muscle mass, strength, and cardiovascular fitness [20,21].

To data, conventional elastic band exercise (EBE) programs for older adults are designed to improve muscular strengths through resistance exercises. The potential benefits of aerobic exercise in a conventional EBE program have attracted relatively less attention. The purpose of the present study was to investigate the effects of incremental circuit exercise training using an elastic band and develop an exercise training program that targets both physical strength and cardiovascular fitness in older adults.

## 2. Method

### 2.1. Study Design and Participants

The current study was a randomized controlled trial (RCT) comprised of 27 older adults (13 men, 14 women) recruited from the Senior Welfare Centre, Korea. The 27 participants were chosen from among 180 who indicated that they were not in the habit of exercising and have no psychiatric problems. Participants were excluded from the study if they indicated any physical problems (i.e., hip, knee, ankle joint problems). 153 adults among the sample of 180 were excluded due to the following reasons: (1) hypertension (*n* = 38); (2) heart disease (*n* = 18); other medical problems (*n* = 65); (3) refusal to participate (*n* = 29); (4) drop-out (*n* = 3). Health History Questionnaire (TriHealth, Cincinnati, OH, USA) was translated into Korean by three translators, and the questionnaire and a verbal interview were used as an initial screening tool. Ultimately, 27 older adults without any physical or medical problems were found and included in the present study.

**Figure 1:**
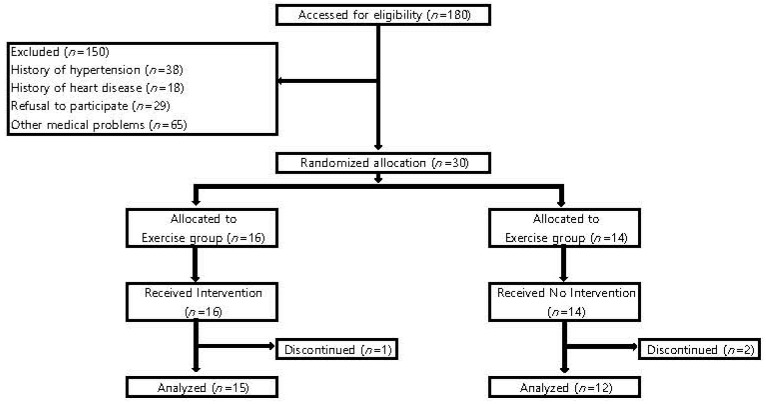
The flow of the randomized controlled study.

Participants were allocated to either the elastic band exercise (EBE) group (EG; *n* = 15) or control group (CTG; *n* = 12) by a randomization technique, the shuffling of playing cards. Participants who picked odd numbers were assigned to the EG, and those who picked even numbers were assigned to the CTG. The study conducted over 12 weeks (Figure 1). All procedures of the study were reviewed and approved by the Institutional Review Board (IRB No. KHU 2014-22), and was performed with the written or verbal consent from the study participants that their demographical and clinical data be extracted and analyzed. Table 1 shows the age, body composition, muscle-fat, blood pressure, and physical activity level of participants in the EG and CTG groups.

### 2.2. Elastic Band Exercise

Generally, elastic band exercises were considered a type of resistance training. The purpose of the present study was to test the effects of conventional circuit training that combined aerobic exercise and resistance exercise using elastic bands.

Participants were transported from the senior welfare centre to a university facility on a bus operated by the centre. Each exercise class lasted for 60 min and three classes were offered per week over the course of 12 weeks. The exercise program was developed based on exercise prescription guidelines for older adults [22,23]. A 60-min exercise consisted of 15-min warm-up, 30-min main exercise, and 15-min cool down [5]. To determine the initial EBE levels for the participants, the rating of perceived exertion (RPE) was measured from each participant while performing elastic band exercises. The purpose of this evaluation was to identify each participant’s medium intensity level, which was to be used as their initial intensity levels. All participants indicated an RPE between 12 and 14 (with 20 being the maximum) when using the red band. Accordingly, the red band (medium intensity) was identified as their initial intensity level. Over the course of 12 weeks, EBE intensity levels were set as follows: (1) for the initial 4 weeks, each exercise was repeated 10 times per session at the red band level (medium); (2) for the subsequent 4 weeks, each exercise was repeated 12 times per session at the red band level (medium); (3) for the final 4 weeks, each exercise was repeated 12 times per session at the green band level (heavy) [24].

The EBE program was composed of an exercise routine starting with upper body exercises and ending with lower body exercises. The 30-min exercise session consisted of front band raises, side lateral band raises, left and right shoulder waves, band hammer curls, band curls, triceps band extensions, triceps band kick-backs, band standing rows, band shoulder rotations, band squats, band dead lifts, standing band triceps extensions, standing band chest presser, upper bands, seated band rows, band leg raises, holding legs props, pelvis relaxations, and band crunches. During each exercise class, an instructor demonstrated each exercise to the participants and two assistants helped the participants follow the exercises. All participants were encouraged to memorize the exercise routine. The study differs from prior EBE studies in that there was no rest allowed between exercise sets (i.e., conventional circuit training). The bands (Hyperform, The Hygenic Corp., Akron, OH, USA) were 30 cm long. When they were stretched 100%, loading weights were 4 kg and 5 kg for the red band and green band, respectively.

### 2.3. Measurement of Body Composition

Height was measured using Martin’s anthropometry (TK-11242, Takei Scientific Instruments Co., Tokyo, Japan). After attaching the equipment to the back of each participant, each participant placed his or her heels evenly and extended their knees. Each participant kept their eyes forward while pulling abdomen and chest. The measurement was recorded in units of 0.1 cm. A digital weighing machine (Tanita, Tokyo, Japan) was used to measure weight without outer clothes and shoes. Weight was recorded in the unit of 0.1 kg. X-scan Plus II (JaWon Medical, Seoul, Korea) in type of bioelectric impendence analysis (BIA) was used to measure fat mass (FM), fat free mass (FFM) to evaluate an indirect index of abdomen fat rate, and body mass index (BMI), respectively.

### 2.4. Measurement of Blood Pressure

Testing blood pressure in a steady state was measured. Each participant took a 20-min rest on a test bed after arriving at the lab. From a sitting position, participants were asked to place an arm parallel to their heart level. A cuff placing a stethoscope on the brachial artery was then affixed. Systolic blood pressure (SBP) and diastolic blood pressure (DBP) were measured twice every 5 min, and the average was used. Mean arterial pressure (MAP) was calculated using the following formula: MAP = [(SBP − DBP) × 1/3] + DBP. Pulse pressure (PP) was also calculated, as follows: systolic blood pressure − diastolic blood pressure.

### 2.5. Measurement of Physical Activity

In the present study, “steps per day” were measured by Trixie accelerometer (Lifecorder, Kenz, Japan) and used as an indirect measure of physical activity. This measure was used only to compare physical activity levels between the two groups in the present study before they entered the 16-week experimental period. Participants were asked to wear the accelerometer all day for 14 days. As previous studies have employed the inclusion criteria of wearing time and days as at least 10 h per day over at least 4 of 7 days, the data were considered acceptable only if participants wore the accelerometer at least 8 days with ≥10 h for each day [25]. Average daily steps were calculated for each participant.

### 2.6. Measurement of Functional Fitness

In this study, functional fitness was measured using simple methods accounting for the physical chanracteristics of the elderly [26].

#### 2.6.1. Muscular Strength

Grip strength was measured to determine muscular strength. While a participant held convenient position with both arms lowered naturally, each participant gripped a dynamometer (GRIP-D 5-100; TAKEI, Tokyo, Japan) and exerted maximum grasping force for 3 s while breathing out. The highest value measured after two tests was used for the study.

#### 2.6.2. Muscular Endurance

Muscular endurance was measured with a Chair Stand Test over a 30-s period. The number of times a participant completed the task was recorded. An instructor demonstrated correct posture and position to every participant prior to measurement. Each participant performed the test twice and the highest value recorded was used for data analysis.

#### 2.6.3. Flexibility

The flexibility was measured by Sit and Reach Test. After sitting subjects forward on a chair with a fixed back, one leg was bent over the floor and another leg was allowed to be straight. It was permitted to easily execute either on left or right leg, but the same leg in the preliminary test was used to measure in the post test. The case of another leg bent for measuring the flexibility conducted another measurement after correcting a position again and the high value was used for the result after performing measurements twice. 

#### 2.6.4. Balance

Static balance sense and dynamic balance sense were used to measure the balance sensory function. Static balance sense refers to the body’s ability to sustain the body in a standing position against gravity on a fixed supporting surface. A one-leg stance with eyes open was used in this study. By folding one leg at a 90 degree angle with eyes open, subjects were tested to measure their ability to sustain feet and arms in a standing position. Dynamic balance refers to the ability to control the body’s position while the body moves without falling down. This study employed the timed up and go (https://www.cdc.gov/steadi/pdf/TUG_test-print.pdf). After setting a target located 2.44 m in front of a fixed-back chair (42 cm tall), the time for walking around it was measured. All balances measurements were taken twice with higher of the two values being used for this study.

### 2.7. Statistical Analysis

SIGMA STAT version 13.0 (Systat Software, Inc., San Jose, CA, USA) was used for a statistical analysis. An independent *t*-test was conducted to confirm physical features of the two groups, two-way repeated measures Analysis of Variance (ANOVA) and Tukey’s post-hoc test were used to verify the interaction between groups and exercising time. In addition, multiple comparison analysis was implemented to verify average differences according to exercising time, and the significant level of all statistical analysis was determined from 0.05 of *p*-value. An appropriate sample size was calculated by using G*power 3.1.9.2. (Heinrich-Heine-Universität Düsseldorf, Düsseldorf, Germany); effect size of 0.30, significance level of 0.05, and power of 0.95. A minimum of 10 study participants was required. Due to the possibility of drop-outs, additional participants were recruited at the beginning.

## 3. Results

Of the 30 individuals enrolled in the study, 27 completed the three months follow-up (97%). Fifteen participants from the EG (80%) and 12 from the CTG group (78%) completed assessment after the intervention (15 in the EG, 80% and 12 in the CTG group, 78%). 

Mean adherence to the exercise program in the EG, for the 78% participants, was 76%, and 30 participants displayed a greater than 75% adherence to the exercise program. The three drop-outs decided to quit the program because of their personal reasons. The groups showed significant differences in SBP (*F* = 8.240, *p* < 0.01), DBP (*F* = 10.454, *p* < 0.01), MAP (*F* = 11.720, *p* < 0.01), and PP (*F* = 7.384, *p* < 0.05) (Table 2). There were significant differences in muscular strength (*F* = 5.490, *p* < 0.05), flexibility (*F* = 4.250, *p* < 0.05), and balance (*F* = 7.888, *p* < 0.01) while no difference in chair stand and timed up and go were found (Table 3).

## 4. Discussion

The present study developed a 12-week EBE program at moderate intensity for elderly adults, and evaluated the effects of the program on physical functions and blood pressure parameters. After 12 weeks, the program had positive effects on SBP, DBP, MAP, and PP. In addition to blood pressure responses, the EBE program also improved muscular strength, flexibility, and static balance in the elderly. This study suggests that the current EBE program with a circuit training design incorporated has positive effects on functional as well as cardiovascular fitness.

### 4.1. Blood Pressure Responses

It is well documented that high-intensity exercise or high-intensity interval training (HIIT) has a positive impact on cardiorespiratory function via numerous factors such as reduced oxidative stress and inflammation, persistent shear stress in vascular endothelium, etc. [26]. Recently, research has focused on moderate-intensity exercise as a potential means of improving cardiovascular fitness for individuals with cardiovascular dysfunctions [27,28]. This research has examined whether physically impaired individuals or older adults may benefit from moderate intensity exercise.

In the present study, resting SBP, DBP, and MAP, were significantly reduced by ~5.0 mmHg after a 12-week EBE program. These results are similar to those of previous studies [29,30], which have shown improvement in the blood pressure of elderly women with high blood pressure after their participation in an elastic band exercise program composed of moderately intense resistance training. Previous studies have shown a positive correlation between cardiovascular diseases and an increase in PP [29,30,31,32]. It follows that the reduction in PP seen in those participants who followed the current EBE program in the present study may reduce cardiovascular disease in older adults. Moderately intense resistance exercises improve vascular endothelial function, thereby reducing arterial contraction and blood pressure [29,30,31,32]. The reduced blood pressure at rest after 12 weeks of EBE, as measured in the present study, could be a result of decreased sympathetic nerve activity [30,33].

### 4.2. Functional Fitness

Exercise training that uses an elastic band is shown to improve functional activities, joint diseases, and physical performance [10,11,12,24]. Remarkably, a previous clinical trial has revealed that home-based exercise training using an elastic band would be effective in improving muscular function, walking function, range of motion at the joint, and perceived pain in patients with osteoarthritis [34]. Other studies have suggested that aerobic exercises combined with resistance training are an important tool for improving mobility impairments and reducing pain in older adults with arthroplasty and osteoarthritis [35,36].

The results of the present study suggested that grip strength could be enhanced after 12 weeks of EBE. Grip strength represents grasping power and the maximum muscular strength of forearms. In a prior study [24], older adults performed resistance exercises using an elastic band and dumbbell for 4 weeks, and their grip strength increased as a result. This improvement may be the result of repeated stimulation of muscles and nerves in the hands, as EBE and dumbbell exercises require repeated acts of grasping, pulling, and relaxing [24]. Muscular mass continues to decrease with advancing age, and can be reduced by as much as 55% in adults in their 70s as compared to their weight in their 30s [31,33]. During the chair stand test in the present study, muscular mass significantly increased after 12 weeks. This suggests that exercises like squats and dead lifts, as part of an EBE program, could strengthen the muscular endurance of the lower legs. This result is consistent with previous studies [12,37] that employed an EBE program for older adults during 8 weeks. 

Flexibility refers to the range of movement of one or many joints. Range of motion is influenced by the structure and function of tissues connected to bones and muscles [38,39]. With advancing age, a lack of physical activity may increase the size of connective tissues (i.e., collagen fibres) of the ligaments and tendons, resulting in decreased range of motion [38,39]. Performing flexibility exercises or stretching exercises on a daily basis improve joint flexibility and prevents muscle shortening [40]. This may lead to enhancement of the flexibility in spondyloarthritis sufferers, as observed in the sit up and reach forward abilities of participants in the present study. Consistent with previous studies [12,37,38,39], sit and reach significantly improved following the EBE program. Lee and Han [37], as well as Barbosa et al. [41], utilized an elastic band exercise for 8 weeks, and resistance exercise for 10 weeks. The flexibility of the older adult participants in the present study improved because the flexion and introversion-type exercises of the lower extremities performed while sitting, as well as the repetitive warm-up, cooling down, and stretching activities.

The balance sensory function indicates physical stability, as controlled by neuro-musculo-skeletal integrations. Studies have shown that women have poorer balance than men and tend to be injured more seriously than men when they fall due to their lower ratio of muscle to body mass [12,38]. Static balance ability measures the body’s control in a standing position against the gravity on the fixed supporting surface, while dynamic balance ability measures the control of position while the body moves. A decline in balance ability for older adults may lead to a decrease of independent physical activity, increasing the risk of falls and the fall-related injuries [12,38]. Participants in the present study showed significant increases in the one-leg stand test, and the timed up and go test also showed a significant decrease. This result is consistent with a study that focused on muscular strengthening of knee joints using an elastic band over 5 weeks [33] and another that tested an EBE program over 4 weeks [12]. Lower extremity muscle strength is closely related to balance [42,43]. In the present study, the tested EBE program improved lower limb muscle strength and flexibility, and consequently improved balancing in older adults. Thus, the elastic band resistance exercise program tested may be considered an effective means of improving physical fitness and health among the elderly.

In the present study, although the intervention was determined to be effective, there were limitations. The initial level of exercise intensity was determined by a subject scale, RPE, which has been well correlated with other golden standards such as %VO_2_max, %1RM, and heart rate reserve [44]. Considering potential health risks of alternative measurement techniques, as well as future applicability of our results as a class-based or home-based exercise intervention, RPE scale was chosen in the present study.

The present study measured resting blood pressure to evaluate cardiovascular fitness. Although stroke volume, cardiac output, endothelial function, and maximal oxygen consumption were also valid indexes to take this measurement, elevated blood pressure is strongly associated with future development of cardiovascular diseases such as stroke, hypertension, and coronary heart disease [45]. Although profound cardiovascular profiles were not measured, findings of the current study showed promise that the exercise interventions can decrease cardiovascular risks for the elderly.

## 5. Conclusions

Elastic band exercises combined with a circuit training component improve both cardiovascular and functional fitness in elderly participants. To benefit the senior citizen community, exercise programs incorporating this insight should be designed and distributed to public health centers and senior citizens homes.

## Figures and Tables

**Table 1:** Physical and physiological characteristics of subjects.

Variables	EG (*n* = 15)	CTG (*n* = 12)	*p*
Age (years)	75.1 ± 1.4	72.3 ± 1.4	0.060
Fat mass (kg)	19.4 ± 1.5	21.3 ± 0.8	0.059
Fat free mass (kg)	24.1 ± 1.1	26.8 ± 1.3	0.062
Body mass index (kg/m^2^)	25.3 ± 0.5	25.5 ± 0.4	0.088
Systolic blood pressure (mmHg)	126.8 ± 2.7	130.4 ± 3.3	0.163
Diastolic blood pressure (mmHg)	78.7 ± 1.9	78.6 ± 3.1	0.456
Physical activity (steps)	5414.1 ± 2.3	5368.3 ± 2.5	0.487

All values were expressed as mean ± standard error; EG = Exercise group, CTG = Control group.

**Table 2:** Changes of blood pressure responses between exercise and control group in EBE program.

Variables	Groups	Pre-Test	Post-Test	∆(%)	*F*	*p*
Systolic blood pressure (mmHg)	EG (*n* = 15)	126.8 ± 2.2	121.3 ± 1.7 ^#,†^	−5.5	8.240	0.006 *
	CTG (*n* = 12)	130.4 ± 3.3	135.7 ± 2.8	4.1		
Diastolic blood pressure (mmHg)	EG (*n* = 15)	78.7 ± 1.7	75.3 ± 1.9 ^#,†^	−5.0	10.454	0.003 *
	CTG (*n* = 12)	78.6 ± 3.1	80.2 ± 2.3	2.0		
Mean arterial pressure (mmHg)	EG (*n* = 15)	95.7 ± 1.5	90.8 ± 1.6 ^#,†^	−5.2	11.720	0.001 *
	CTG (*n* = 12)	96.5 ± 3.1	100.0 ± 2.0	3.6		
Pulse pressure (mmHg)	EG (*n* = 15)	51.5 ± 2.0	46.9 ± 1.9 ^#,†^	−8.9	7.384	0.010 *
	CTG (*n* = 12)	53.7 ± 1.4	53.5 ± 3.2	−0.4		

* Statistically significant. Values were expressed as mean ± standard error; this is the interaction (group condition) using repeated measured two-way ANOVA adjusting for sex as covariance. *p* value was calculated using repeated measured two-way ANOVA (group time interaction); EBE = Elastic band exercise, EG = Exercise group, CTG = Control group. ^#^ significantly different vs. pretest, ^†^ significantly different vs. CTG.

**Table 3:** Changes of functional fitness between exercise and control group in EBE program.

Variables	Groups	Pre-Test	Post-Test	∆(%)	*F*	*p*
Grip strength (kg)	EG (*n* = 15)	24.6 ± 1.6	28.3 ± 2.1 ^#,†^	14.9	5.490	0.026 *
	CTG (*n* = 12)	21.7 ± 1.2	22.2 ± 1.2	2.7		
Chair stand (count/30 s)	EG (*n* = 15)	14.2 ± 1.3	17.8 ± 1.3 ^#^	25.4	2.197	0.149
	CTG (*n* = 12)	15.8 ± 1.0	17.3 ± 0.8	10.1		
Sit and reach (cm)	EG (*n* = 15)	8.9 ± 1.7	11.0 ± 1.7 ^#,†^	24.8	4.250	0.048 *
	CTG (*n* = 12)	15.3 ± 2.3	15.0 ± 1.7	−1.5		
One-leg stance (s)	EG (*n* = 15)	26.0 ± 6.4	33.8 ± 5.9 ^#,†^	29.8	7.888	0.009 *
	CTG (*n* = 12)	22.4 ± 5.5	21.6 ± 5.4	−3.8		
Timed up and go (s)	EG (*n* = 15)	12.2 ± 3.5	6.9 ± 0.5	−43.5	2.275	0.142
	CTG (*n* = 12)	8.1 ± 0.7	8.0 ± 0.6	−1.8		

* Statistically significant. Values were expressed as mean ± standard error; this is the interaction (group condition) using repeated measured two-way ANOVA adjusting for sex as covariance. *p* value was calculated using repeated measured two-way ANOVA (group time interaction); EBE = Elastic band resistance exercise, EG = Exercise group, CTG = Control group. ^#^ significantly different vs. pretest, ^†^ significantly different vs. CTG.
